# A comparison of 0.375% ropivacaine psoas compartment block and 2% prilocaine spinal anaesthesia in dogs undergoing tibial plateau levelling osteotomy

**DOI:** 10.1186/s12917-022-03277-6

**Published:** 2022-05-12

**Authors:** Diego Sarotti, Elena Lardone, Lisa Piras, Davide Mancusi, Paolo Franci

**Affiliations:** 1 Centro Veterinario Fossanese, Via Cuneo 29/E, 12045 Fossano, CN Italy; 2 Department of Veterinary Science, University of Turin, Largo Paolo Braccini 2, 10095 Grugliasco, Italy

**Keywords:** Same-day surgery, Spinal anaesthesia, Peripheral block, Prilocaine, Morphine, Dog

## Abstract

**Background:**

In dogs undergoing routine elective orthopaedic surgeries carried out as same-day surgeries regional anaesthetic techniques (RATs) should aim to produce analgesia but minimising the postoperative motor dysfunction. Our objective was to compare the perioperative analgesic effects and the time to motor recovery between spinal anaesthesia (SA) with hyperbaric solution of prilocaine 2% (mg = 4 x [0.3 × BW (kg) + 0.05 × SCL (cm)]) and morphine (0.03 mg/kg) and combined ultrasound (US) and electro stimulator-guided psoas compartment and ischiatic nerve block (PB) with ropivacaine 0.375% (0.45 mL/kg). Dogs undergoing tibial plateau levelling osteotomy (TPLO) were randomly assigned to receive either SA or PB. Procedural failure, perioperative rescue analgesia, motor block recovery and complications were recorded.

**Results:**

Procedural failure rate (PFR) was 19% (7 out of 36) for SA and 9% (3 out of 32) for PB (*p* = 0.31). Intraoperative rescue analgesia was administered to 6/29 (21%) SA group dogs and in 15/29 (52%) PB group dogs, respectively (*p* = 0.03). At 3 h after RAT, percentage of dogs with complete block recovery was 25/29 (86%) and 25/29 (86%) in group SA and PB, respectively (*p* = 1). Two cases of pruritus and one case of urinary retention were recorded in the SA group. Residual ischiatic nerve block was noted at 12 h after RAT in 2/15 (13%) of dogs in group PB; it completely resolved 24 h after RAT.

**Conclusions:**

SA with prilocaine 2% and PB with ropivacaine 0.37% were found suitable for dogs undergoing same-day TPLO surgery. Pruritus and urinary retention in SA and residual block in both groups might occasionally delay the time of discharge.

**Supplementary Information:**

The online version contains supplementary material available at 10.1186/s12917-022-03277-6.

## Background

In humans, same-day routine elective orthopaedic surgeries can offer several advantages [1, 2]: it relieves the night shift team of the burden of prolonged hospitalization, leaving more time for demanding cases, minimizes the stress on the patient in what is often perceived as an unfriendly environment, and lowers the risk of cross-sectional infection [3, 4]. Not every veterinary practice has the possibility of guarantee overnight hospitalization, therefore elective routine orthopaedic surgeries, such as tibial plateau levelling osteotomy (TPLO), could also be performed as same-day surgery in dogs. This might also reduce costs and waiting lists.

Day surgery and related clinical research have attracted increasing interest in human medicine [5] but scarce attention in veterinary one.

Anaesthesia for same-day surgeries must ensure rapid onset and off-set, rapid postoperative mobility, and optimal control of postoperative pain. The use of regional anaesthetic techniques (RATs) could meet these requisites. In particular, unilateral intrathecal anaesthesia and peripheral nerve blocks (PB) could be RATs of choice because of their rapid onset, predictable duration of sensory and motor block with minimal side effects [6, 7].

In dogs undergoing orthopaedic surgeries, intrathecal anaesthesia with bupivacaine was extensively investigated, but because the postoperative motor recovery time was prolonged [8, 9], it would not be a satisfactory choice for a same-day surgery. Hyperbaric prilocaine 2%, which was licensed for spinal anaesthesia (SA) in Europe in 2011, is a short-acting drug that fulfils the key criterion of an ideal intrathecal agent for same-day surgery [10, 11].

The aim of this double-center prospective randomized clinical study was to compare the perioperative analgesic effects and the time to motor recovery between unilateral SA (hyperbaric solution of prilocaine 2% and morphine 1%) and psoas compartment and ischiatic nerve block performed with the aid of an US-guided and a nerve stimulator technique (local anaesthetic ropivacaine 0.375%) in dogs undergoing TPLO surgery for rupture of the cruciate ligament. The primary hypothesis was that SA should reduce the nociceptive response to surgery as consequence, the number of intraoperative rescue analgesia administrations compared to PB. Our secondary hypothesis was that there was no difference between SA and PB regarding postoperative rescue analgesia and motor block recovery.

## Methods

This trial was reviewed and approved by the Ethical Committee of the Department of Veterinary Science – University of Turin, Italy (Prot. No. 1515/2020). Written informed consent was signed by the owners at dog admission. Dogs scheduled for TPLO between July 2020 and April 2021 at the Veterinary Teaching Hospital of Turin (Italy) and at Centro Veterinario Fossanese (Fossano, Italy) were recruited. All dogs underwent preoperative physical examination and blood analysis. Exclusion criteria were: American Society of Anesthesiologists classification (ASA) > 2, age < 6 months, infectious skin disease affecting the lumbosacral area, history of bleeding disorders, uncorrected hypovolemia, neurodegenerative central or peripheral diseases, and anatomical spinal abnormality. Two treatment groups SA group and PB group were formed according to simple randomization based on a computer-generated randomization sequence (www.randomizer.org). The estimated sample size to detect a difference in the primary endpoint (power 80% and alpha error 5%) assuming an intraoperative rescue anaesthesia (iRA) of 20% in the SA and 50% in the PB group, respectively, was 36 dogs per group (https://select-statistics.co.uk/calculators/sample-size-calculator-two-proportions) [12]. Accounting for possible dropouts, we decided to enroll 40 dogs per group and to plan an interim statistical analysis after enrollment of at least 60 dogs. At this stage, if a clear superiority of one of the two groups would have been found the study would have been interrupted.

**Fig. 1:**
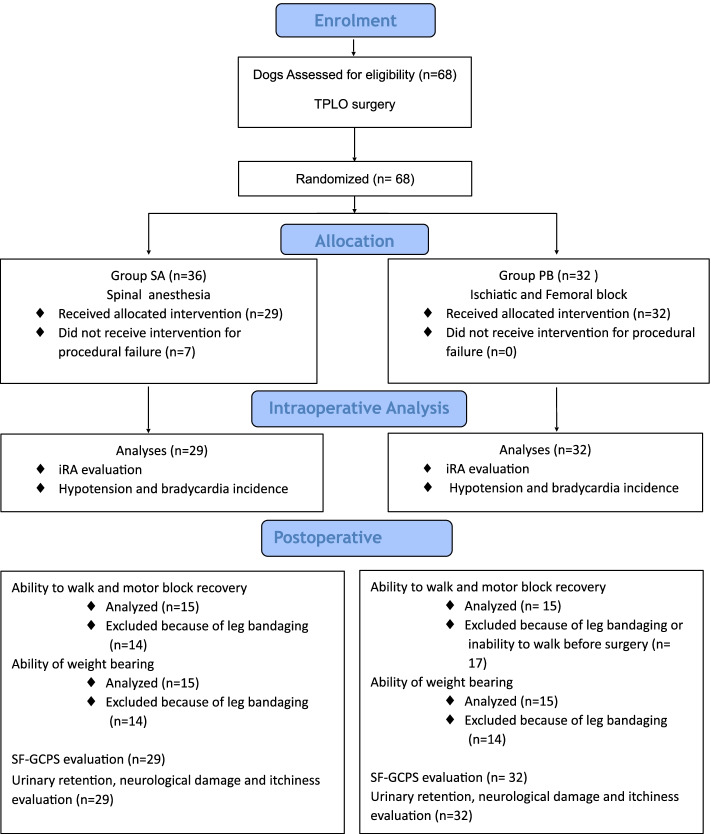
Consolidated Standards of Reporting Trials flow diagram. Intraoperative rescue analgesia (iRA), Short Form Glasgow Composite Pain Scale (SF-GCPS)

The manuscript conforms to the Consolidated Standards of Reporting Trials (CONSORT) Statement 2010 for reporting randomized clinical trials [13] (Fig. 1). SA and PB were performed by two experienced operators (PF, DS), other two anaesthetists (EL, MS) blinded to treatment monitored the dogs during surgery. All surgical procedures were performed by two surgeons (LP, DM) who were unaware of the assigned group.

### Anaesthesia protocol and procedures

Dogs of group PB received an intramuscular (IM) injection of dexmedetomidine (1 μg/kg, Dexdomitor; Orion) and methadone hydrochloride (0.2 mg/kg, Semfortan; Dechra) as pre-anaesthetic medication. In group SA, only dexmedetomidine (1 μg/kg, Dexdomitor; Orion) was used. The cephalic vein was catheterized and a volume of 5 mL/kg/h of lactated Ringer’s solution (lactated Ringer’s; Fresenius Kabi) was administered intravenously (IV). Intravenous propofol (Proposure 1%; Merial) was titrated to effect to induce general anaesthesia. After orotracheal intubation, isoflurane (Isoflo; Esteve) was delivered in a mixture of medical air and oxygen with a fraction of inspired oxygen of 0.4, using a rebreathing system. The dorsal pedal artery was cannulated with a catheter of suitable dimension (Surflo; Terumo), pressure transducer was zeroed at the level of dog’s sternal manubrium. Intraoperatively, a multiparametrical monitor (Infinity Delta; Draeger or Datex AS3, Draeger) was used to monitor cardiovascular (systolic, diastolic, mean arterial pressures [SAP, DAP, MAP], heart rate [HR] and rhythm) and respiratory (end tidal carbon dioxide [P_E_’CO_2_], peak inspiratory pressure [PIP], respiratory rate [*f*_R_], tidal volume [V_T_], minute volume [V_E_], inspired fraction of oxygen [FiO_2_], end tidal isoflurane fraction [F_E_’_ISO_]) parameters, as well as esophageal temperature (T,°C). Perioperatively, an active heating system (Bair Hugger Warmer Model 505; Augustine Biomedical Design) was used to maintain a body temperature above 36 °C. Volume-controlled intermittent positive pressure ventilation (Cato; Draeger, or Primus; Draeger) was applied to maintain values of P_E_’CO_2_ between 35 and 40 mmHg. All data were manually recorded every 5 min (min) for the entire duration of anaesthesia. The time to perform RATs was defined as the minutes between the end of surgical preparation and injection of the LA solution (for PB) or the end of 10-min fixation of LA (for SA). The number of attempts was also recorded. Each new skin puncture was recorded as another attempt.

### SA group

Dogs were positioned in lateral recumbency with the limb to be operated lowermost. Both hind limbs were extended cranially and kept parallel by an assistant. The ilium wings, together with the dorsal spinous process of L6, were used as anatomical landmarks. After surgical preparation of the skin, the subarachnoid space was reached inserting a Quincke needle (22, 23 or 25 gauge; Becton, Dickinson and Company) on the dependent side using a paramedian approach and with the bevel facing cranially. The needle was directed from the lateral to the cranial aspect of the spinous process of L6 vertebra, until it was in contact with the lamina. The needle was then advanced cranioventrally toward the midline until the intervertebral space was identified and then forwarded through the subarachnoid space. Proper needle placement was ascertained by detection of cerebrospinal fluid in the needle hub. A hyperbaric solution of prilocaine hydrochloride (Prilotekal 2%; B Braun) and morphine hydrochloride (Morphine hydrochloride 1%; Molteni) was injected as a single bolus at a rate of about 1 mL per min.

The prilocaine dose was calculated based on body weight (BW) and spinal cord length (SCL) as described by Sarotti et al. (2013) and assuming that bupivacaine is four times more potent than prilocaine [14]:

$$\mathrm{Prilocaine}\ \left(\mathrm{mg}\right)=4\times \left[0.3\times \mathrm{BW}\ \left(\mathrm{kg}\right)+0.05\times \mathrm{SCL}\ \left(\mathrm{cm}\right)\right]$$

The morphine dose calculated in relation to BW was 0.03 mg/kg.

SA was performed by two experienced anaesthetists (DS, PF). The number of attempts and the time to perform SA was recorded. After injection the dog was kept in the same lateral recumbency for 10 additionally minutes. This fixation time was considered as part of the time taken to perform SA. After three attempts without cerebral spinal fluid outflow, the procedure was aborted and recorded as a procedural failure. Each new skin puncture was recorded as another attempt.

### Peripheral Block group (PB)

Psoas-ischiatic nerve blocks were performed combining ultrasound- and nerve stimulator-guided (Stimuplex HNS 12; B Braun) techniques. Ultrasound guidance was performed using a high-frequency 12 MHz linear array transducer (HS 50; Samsung or Mylab 30 gold; Esaote).

The dogs were positioned in lateral recumbency with the limb to be operated uppermost. The anatomical site was then aseptically prepared and the positive electrode for stimulation was applied to the skin of the ventral abdomen.

Ropivacaine (Naropina® 0.75%; Fresenius Kabi) was diluted with an equivalent volume of 0.9% saline solution (Sodium chloride 0.9%; Fresenius Kabi) to obtain a 0.375% ropivacaine solution. A syringe containing 0.45 mL/kg of 0.375% (equivalent to 1.69 mg/kg) ropivacaine was aseptically prepared and connected to an insulated stimulating needle of appropriate length (Stimuplex D; B Braun).

### Psoas compartment block (femoral and obturator nerve block)

The iliopsoas muscle was sonographically identified using an in-plane technique, as suggested by Tayari et al. (2017) [15]. When the psoas muscle was visualized, an insulated stimulating needle was inserted cranially to the iliac wing and directed caudomedially a pulse width of 0.1 ms and a stimulation frequency of 2 Hz. The needle was advanced until a contraction of the quadriceps muscle and extension of the stifle were evident [16] and maintained with a current ≥ 0.4 but ≤ 0.6 mA. When contraction was not achieved, the needle was redirected toward other hypoechoic structures. If the electro locator failed to identify where the femoral nerve was, the LA was injected into the area where the nerve was expected. After negative aspiration of blood to rule out intravascular needle placement, a volume of 0.3 mL/kg of 0.375% ropivacaine was injected in the psoas muscle. Any time the femoral nerve was not found by ultrasound nor was the muscle contraction elicited with the nerve locator was recorded as a procedural failure event.

### Ischiatic nerve block

An US-guided nerve block of the ischiatic nerve was performed using a medium lateral approach by placing the probe on the lateral aspect of the proximal third of the thigh. When the ischiatic nerve was identified in the fascia between the adductor muscle and the biceps femoris muscle, an insulated stimulating needle was inserted in-plane in caudo-cranial direction through the semimembranosus muscles and advanced until contraction of the *tibialis cranialis* or *gastrocnemius* muscle was evident. When contraction was maintained with a current ≥ 0.4 but ≤ 0.6 mA and negative aspiration of blood was detected, 0.15 mL/kg of 0.375% ropivacaine was injected.

### Intra-operative evaluation and treatment

The duration of both surgery and anaesthesia (min) were recorded as it was the incidence of bradycardia (HR < 60 beats/min with a MAP < 60 mm Hg for at least 5 min) and hypotension (MAP < 55 mm Hg). Hypotension was treated by reducing by 20% the F_E_’_ISO_ and giving a 3 mL/kg bolus of lactated Ringer’s solution IV in 60 s (sec). If MAP increased after the first bolus, an additional 2 mL/kg of fluid in 60 s was administered. If hypotension persisted, a bolus of ephedrine (50–100 μg/kg) and/or a continuous rate infusion of norepinephrine (0.1–0.3 μg /kg/min) was given [17].

Intraoperative rescue analgesia (iRA) (fentanyl 1 μg/kg IV) was administered if the MAP rose by more than 30% of the pre-incisional level, which was defined as the mean pressure measured during the 5 min prior to skin incision. The fentanyl bolus (1 μg/kg IV) was repeated every 3 min until the MAP returned to the pre-incisional level. The iRA incidence and the number of fentanyl boluses between the two groups were compared [8].

Arousal events were defined as intraoperative movement, brisk palpebral reflex, or spontaneous breathing against mechanical ventilation. In such cases propofol 1 mg/kg was administered IV. At the end of surgery, IV meloxicam (0.2 mg/kg, Metacam; Boehringer) was administered to all dogs and the urinary bladder was voided manually [8, 17].

### Postoperative evaluation and treatment

Two experienced operators (LP, DM) unaware of the treatment evaluated each dog during the first 24 h after RAT. Quality of recovery from general anaesthesia was scored using a recovery scoring system [18] (Additional file 1: Appendix 1A).

Postoperative pain was assessed using the Short Form of the Glasgow Composite Pain Scale (SF-GCPS) every 2 h starting from 3 h after RAT [19]. Methadone (0.1 mg/kg) was administered IM if the pain score was ≥ 5/20. The dogs were re-evaluated 30 min later and a further methadone dose (0.1 mg/kg IM) was administered if needed. The number of rescue methadone administrations during the first 24 postoperative h was recorded. A previously published weight-bearing scoring system was used before (T0) and 24 h after surgery (T24) [20] (Additional file 2: Appendix 2A).

The assessment of the complete motor block recovery was performed at 3, 5, 8, 12, and 24 h after RAT by an operator unaware of the RAT technique (LP, DM). Dogs had to walk on their own, but they were assisted to stand up, if necessary. Dogs that were unable to walk before surgery or that had postoperative bandaging of the leg were not assessed.

Urinary retention was defined as the inability to spontaneously void in the presence of bladder overdistension. If a dog did not spontaneously urinate within 12 h after local anaesthetic injection, both abdominal palpation and US scan were performed to evaluate bladder overdistension [8, 17].

### Statistical analysis

Categorical variables are reported as frequency and percentage; Fisher’s exact test was used to evaluate frequency distribution independence between the two groups. The Lilliefors test was performed on continuous variables to check for normal distribution. Not normally distributed data are reported as median and range and were analysed using the Mann–Whitney U test. Comparison of median weight-bearing scores among different time points (T0 vs T24) within the same group were performed using Friedman's test. Statistical analysis was performed using MedCalc Software for Windows version 12.5 (MedCalcSoftware, Ltd., Belgium). Significance was set at 5% for all statistical methods.

## Results

The study was conducted on 68 dogs: 36 were assigned to group SA and 32 to group PB. Procedural failure was recorded in 7/36 (19%) in SA group and in 3/32 (9%) in PB group dogs (*p* = 0.31), respectively. The femoral nerve could not be clearly identified by ultrasound in 8/32 (25%) PB group dogs.

There was no difference in median weight, age, ASA status, and type of surgery between the two groups (Table 1). Table 1 presents procedural data for the median propofol bolus for induction, median F_E_’_ISO_ (%) during surgery, time taken to perform the locoregional technique, time between induction and LA injection (min), time between LA injection and skin incision (min), time between LA injection and end of the operation (min), entire duration of anaesthesia (min), and cases with at least one arousal event (%).

**Table 1:** Demographic and procedural data for the SA group and the PB group. Median F_E_’_ISO_ (%) during surgery is higher in PB Group compared with SA Group (*p* = 0.01). Median time to perform SA (min) considers the 10 min required for fixation of the local anaesthetic

	SA Group (*n* = 29)	PB Group (*n* = 29)	*p*-value
Breed (n)	14 Mix breed	9 Mix breed	Not evaluated
	3 Labrador retriever	6 Labrador retriever	
	2 Jack russel	2 Jack russel terrier	
	2 Maremma sheepdog	2 Boxer	
	1 Yorkshire terrier	1 Golden retriever	
	1 Boxer	1 Cavalier King Charles Spaniel	
	1 Bloodhound	1 Border collie	
	1 Australian shepherd	1 Malinois	
	1 Maltese	1 American Staffordshire	
	1 Argentinian dogo	1 Volpino	
	1 Poodle	1 Cocker	
	1 Bernese mountain dog	1 Leonberger	
		1 German shepherd	
		1 Cane corso	
Age (years)	7 (0.8–10)	7 (1–12)	0.29
Weight (kg)	23.5 (4–56)	24 (5–56)	0.45
Type of surgery (n)	Tibial plateau levelling osteotomy (TPLO) without arthrotomy: 15	Tibial plateau levelling osteotomy (TPLO) without arthrotomy: 16	0.22
	Tibial plateau levelling osteotomy (TPLO) with arthrotomy: 14	Tibial plateau levelling osteotomy (TPLO) with arthrotomy: 13	
ASA Class (n)	ASA I: 27/29 (93%)	ASA I: 28/29 (97%)	0.6
	ASA II: 2/29 (7%)	ASA II: 1/29 (3%)	0.6
Median propofol induction bolus (mg/ kg)	5 (2.4–10.8)	4 (3.3–7.5)	0.38
Median FE’ISO of isoflurane (%) during surgery	1.2 (0.8–1.4)	1.3 (0.9–1.4)	0.01
Median time to perform locoregional technique (min)	16 (12–32)	10 (4–15)	< 0.001
Median time between induction and local anaesthetic (LA) injection (min)	33 (22–62)	36 (25–45)	0.91
Median time between LA injection and skin incision (min)	28 (17–34)	25 (12–37)	0.08
Median time between LA injection and end of surgery (min)	92 (73–103)	105 (72–135)	0.06
Median duration of entire anaesthesia (min)	140 (119–172)	147 (120–188)	0.18
Cases with at least one arousal event (%)	0/29 (0)	0/29 (0)	1

The CONSORT diagram shows the number of dogs entered in the intraoperative and the postoperative analysis (Fig. 1).

Hypotension developed in 10/29 (35%) SA group and in 6/29 (21%) group PB dogs, respectively (*p* = 0.38) and bradycardia in 2/29 (7%) group SA and in 9/29 (31%) group PB dogs, respectively (*p* = 0.04). The median MAP during surgery was 73 (55–104) mm Hg in the SA group and 82 (70–99) mm Hg in the PB group (*p* = 0.01). The median heart rate during surgery was 80 (58–110) beat/min in the SA group and 76 (50–117) beat/min in the PB group (*p* = 0.22).

The overall iRA incidence was 6/29 (21%) in the SA group and 15/29 (52%) in the PB group, respectively (*p* = 0.03). The median amount (mcg/kg) of fentanyl administered during surgery was 0 (0–2) in the SA group and 1 (0–4) in the PB group (*p* < 0.01). Table 2 presents the recovery quality score, the number of dogs requiring pRA and the median weight-bearing score before (T0) and 24 h after surgery (T24).

**Table 2:** Postoperative evaluation. There was no difference in recovery quality and postoperative rescue analgesia (pRA) between the two groups. Percentage of dogs with complete block recovery was statistically significant different at 3, 5, and 8 h

Criterion	SA Group	PB Group	*p*-value
Recovery quality score	0 (0–2)	0 (0–2)	0.17
Dogs pRA requiring (%)	0/29 (0%)	2/29 (12%)	0.40
Median weight-bearing score at T0	3 (1–4)	2 (1–4)	0.90
Median weight-bearing score at T24	2.5 (1–4)	2 (1–4)	0.51
Complete block recovery at 3 h (%)	13/15 (87%)	5/15 (33%)	0.008
Complete block recovery at 5 h (%)	14/15 (93%)	7/15 (47%)	0.014
Complete block recovery at 8 h (%)	15/15 (100%)	9/15 (60%)	0.017
Complete block recovery at 12 h (%)	15/15 (100%)	13/15 (87%)	0.48
Complete block recovery at 24 h (%)	15/15 (100%)	15/15 (100%)	1

Residual block of the ischiatic nerve at 12 h was noted in 2/15 (13%) PB group dogs, which completely resolved at 24 h post-surgery

Two cases of pruritus and one case of urinary retention were recorded in the SA group. The two episodes of pruritus involved the back area at around 30 min after extubation and was treated with two boluses of propofol (1 mg/kg)

The median weight-bearing score within group was not different between T0 and T24 (in SA Group *p* = 0.73 and in PB Group *p* = 0.63).

## Discussion

SA and psoas compartment and ischiatic nerve block ensured sufficient control of intraoperative nociceptive stimuli, rapid postoperative motor recovery, and prolonged postoperative analgesia. Therefore, the two RTAs used could be implemented in dogs undergoing an orthopedic procedure in same-day elective routine orthopaedic surgeries. However, SA during the intraoperative period achieved better quality of nervous block. As widely reported [21], SA causes a deep and homogeneous nervous block, which may have substantially contributed to the low need for iRA and fentanyl consumption in the SA group.

To the best of our knowledge, this is the first study to use prilocaine for SA in dogs. Prilocaine is a short-acting amino-amidic local anaesthetic agent which has been mainly studied in humans for SA in patients undergoing various surgical procedures in a day surgery regimen. It shares characteristics with lidocaine (rapid onset and offset) but has a much better safety profile (lower neurotoxicity) [22]. The later characteristic allows the administration of prilocaine solution, made hyperbaric by adding glucose (usually 8%), which produces high concentration of the local anaesthetic around the intervertebral nerve roots on the dependent side, increasing the quality and the duration of the block. In a previous study in dogs receiving SA with hyperbaric solution of bupivacaine for hind limb orthopaedic procedures, the incidence of iRA was similar to our study while the motor block recovery was faster in our study using prilocaine [14]. In another study using an equivalent dose of bupivacaine, 85% of dogs were reported able to walk at 5 h [8]. Comparison of the two studies shows that the use of hyperbaric prilocaine provided a similar quality of nervous block, with a shorter duration of motor impairment and early mobilization, which is an important goal in the same-day surgery. The duration of prilocaine block in humans is longer than that of spinal block achieved with other short-action local anaesthetics such as chlorprocaine [23]. Several studies in humans have attempted to find the optimal dose of prilocaine to obtain a more reliable intraoperative block and a faster postoperative discharge [24, 25]. In our study, our choice of the LA dose regimen assumed equipotency between prilocaine 2% and bupivacaine 0.5% [26]; we applied a previously published dose regimen in dogs for bupivacaine.

In our study, more than 50% of the dogs in the PB group received at least one intraoperative dose of fentanyl. This finding is shared by previous studies. Tayari et al. (2017) reported administrations of iRA in 70% of dogs undergoing TPLO after having received psoas compartment and ischiatic nerve block by the US-guided technique [15]. Palomba et al. (2020) reported administrations of iRA in 50% of dogs during a similar type of surgery and blocks using only electrical nerve localization [18]. There are many possible reasons for the higher incidence of iRA in the PB group beyond the recognized difference in the nerve block quality achieved by the two techniques.

Although the femoral nerve was our echo graphic landmark to deposit the local anaesthetic, this compartmental block aims, by using an appropriate volume of local anaesthetic, to reach all the nerves involved in the sensitive innervation of the hind limb. Inclusion of the obturator nerve in the block is relevant for perioperative analgesia, given that it can supplies nervous fibers to the medial articular nerve of the knee joint in more than 10% of subjects [27]. Therefore, US-guided and electrical nerve localization may not be effective for blocking a single nerve. How the local anaesthetic spreads within the psoas muscle could play an important role in the overall block quality and, subsequently, in the incidence of iRA and postoperative pain. The claimed superiority of the US-guided technique is not supported by the present or other studies. It is probably for this reason that the intraoperative incidence of iRA was not improved by using both echography and neurolocalization, as compared to other studies in which only the latter was used [18]. Furthermore, the combined use of US-guided and electrical nerve localization in our study was not always successful. The femoral nerve could not be found by ultrasound nor muscle contraction elicited with the nerve locator in 3 PB group dogs. Ultrasound identification of the psoas compartment is usually easy; however, a clear view of the femoral nerve was not obtained in a quarter of cases, although a muscular contraction was elicited.

In addition, the use of a rather diluted LA may have also played an important role. The volume of the LA is crucial for the psoas compartmental block in order to wet the lumbar plexus. The volume of LA used in the present study was higher than in previous studies [15, 18, 28]. Here we wanted to minimize postoperative motor block and so we used a diluted ropivacaine solution (0.375%) while maintaining a total dose per kg only slightly higher than that reported in other studies [18]. Palomba et al. (2020) used a similar total dose of levobupivacaine per kilogram per block, but the LA solution was more concentrated (0.5%), causing a more prolonged motor block with a median time of unassisted walking of nearly 9 h.

As performed in this study, both RATs, combined with the perioperative administration of a non-steroidal-anti-inflammatory drug, could be adequate to provide analgesia during the first 24 postoperative hours. Our findings agree with a recently published study in which dogs undergoing TPLO were not regularly treated with full mu agonist opioids postoperatively [29]. Therefore, either SA with 2% prilocaine and morphine or psoas compartment block with ropivacaine 0.375% could be considered for same-day TPLO surgery.

Studies in humans have reported that a single-shot psoas compartment block, as part of a multimodal analgesic technique, provided in median verbal rating scores of zero in the first 24 h after total knee arthroplasty [30]. The posterior lumbar plexus (psoas compartment) block and the three-in-one femoral nerve block provide similar postoperative analgesia after total knee replacement in humans. Other comparable studies have reported higher postoperative opioid needs than our study [15, 18]. Palomba et al. (2020) administered opioids to dogs that were non-weight bearing on the operated leg postoperatively; however, such a clinical finding should not be considered an unequivocal sign of severe pain, as it is indirectly suggested by the SF-GCPS that accounts lame walking just with one point.

We noted no difference in the weight bearing score before and at 24 h after surgery. This may be the result of altered biomechanics of the stifle joint, which may condition weight-bearing behaviour during the postoperative period in dogs undergoing TPLO surgery [31].

While SA reduced iRA incidence and fentanyl consumption, the procedural failure rate was quite high. In our study, the operator was unable to perform the spinal technique in almost 1 out of 5 SA group dogs. In addition to the higher incidence of postoperative complications, this is a relevant disadvantage of SA: it wastes time and changes the perioperative anaesthesia planning. When an expert operator performs the block, such a failure is mainly due to difficult positioning of the needle within the vertebral canal, which is especially frequent older animals with spondylarthrosis in which accessing the interspinous foramen may be difficult. Low cerebrospinal fluid pressure within the subarachnoid space can be another potential cause of procedural failure in attempting SA in dogs. Puncturing from the dependent side of recumbency and anti-Trendelenburg positioning of the dog can be useful to facilitate the cerebrospinal fluid outflow [8].

A limitation of the present study is the premedication regimen that differed between the two groups: 0.2 mg/kg of methadone (IM) was administered as premedication in the PB group, whereas 30 mcg/kg of morphine was administered intrathecally in the SA group. Even though dogs in the latter group received a much lower dose of opioid, the magnitude and duration of intrathecal morphine may have produced a relevant sparing effect on intraoperative fentanyl requirements [32].

The combined use of dexmedetomidine and methadone could explain the higher incidence of bradycardia events in the PB group [33]. Sarotti et al. [14] found an incidence of 8% of urinary retention in a group of 39 dogs undergoing hind limbs orthopedic surgery, receiving hyperbaric bupivacaine SA, a much higher amount compared to the incidence found in this study (3%).

For this double centre study, a considerable number of cases were collected in a relatively short period; nevertheless, the multicenter nature of this study may have resulted in high variability of the data. The protocol for anaesthesia and analgesia was the same at the two study centres but the surgeons were different. Arthrotomy was always performed at the one institution but rarely at the other. The two surgeons, while similar in expertise, differed in how they carried out intraoperative surgery stimulation and evaluated postoperative pain. The median F_E_’_ISO_ (%) during surgery was slightly higher in the PB group. This factor could have reduced the iRA in the PB group, though the quality of intraoperative nociception was higher in the SA group.

## Conclusion

SA with hyperbaric solution of prilocaine 2% and morphine and combined US and electro stimulator-guided psoas compartment and ischiatic nerve block with ropivacaine 0.37% were found suitable for undergoing TPLO in day surgery regimen. Pruritus and urinary retention in SA and residual block in both groups occasionally can delay the time of discharge.

## Supplementary Information

**Additional file 1:** **Appendix A1.** Recovery quality scoring system.

**Additional file 2:** **Appendix A2.** Weight-bearing scoring system.

## Data Availability

The datasets used and/or analysed during the current study are available from the corresponding author (EL) on reasonable request.

## Abbreviations

RATs: Regional anaesthetic techniques
SA: Spinal anaesthesia
US: Ultrasound
PB: Peripheral nerve block
PFR: Procedural failure rate
TPLO: Tibial plateau levelling osteotomy
ASA: American Society of Anaesthesiologists
IRA: Intraoperative rescue anaesthesia
CONSORT: Consolidated Standards of Reporting Trials
IM: Intramuscular
IV: Intravenously
SAP; DAP; MAP: Systolic, diastolic, mean arterial pressures
HR: Heart rate
P_E_’CO_2_: End tidal carbon dioxide
PIP: Peak inspiratory pressure
*f*_R_: Respiratory rate
V_T_: Tidal volume
V_E_: Minute volume
FiO_2_: Inspired fraction of oxygen
F_E_’_ISO_: End tidal isoflurane fraction
T: Temperature
Min: Minutes
BW: Body weight
SCL: Spinal cord length
SF-GCPS: Short Form of the Glasgow Composite Pain Scale
pRA: Postoperative rescue analgesia
