# Supplementary material for: A comparison of 0.375% ropivacaine psoas compartment block and 2% prilocaine spinal anaesthesia in dogs undergoing tibial plateau levelling osteotomy
Source: BMC Vet Res. 2022 May 12;18:172. doi: 10.1186/s12917-022-03277-6 (PMC9097125; doi:10.1186/s12917-022-03277-6)
Supplement: Supplementary file 1 — Additional file 1: Appendix A1. Recovery quality scoring system. [file 12917_2022_3277_MOESM1_ESM.docx]

Appendix A1 Recovery quality scoring system. (Palomba et al. 2020)

| Score | Classification | Descriptors |
| --- | --- | --- |
| 3 | Poor | Violent recovery requiring sedation due to excitation, loud vocalization |
| 2 | Acceptable | Mild vocalization, settles if given attention, sedation is not needed on safety grounds but may be administered for smooth recovery |
| 1 | Good | The animal recovers well from anaesthesia, no vocalization, but it appears not fully responsive/interactive, as if it had received sedation |
| 0 | Excellent | The animal recovers from anaesthesia smoothly, as if it were waking up from natural sleep, no sedation is apparent, and the animal is responsive/interactive |
