# Supplementary material for: A comparison of 0.375% ropivacaine psoas compartment block and 2% prilocaine spinal anaesthesia in dogs undergoing tibial plateau levelling osteotomy
Source: BMC Vet Res. 2022 May 12;18:172. doi: 10.1186/s12917-022-03277-6 (PMC9097125; doi:10.1186/s12917-022-03277-6)
Supplement: Supplementary file 2 — Additional file 2: Appendix A2. Weight-bearing scoring system. [file 12917_2022_3277_MOESM2_ESM.docx]

Appendix A2. Weight-bearing scoring system. Modified by Nganvongpanit et al. (2013).

| Score | Classification | Descriptors |
| --- | --- | --- |
| 4 | No bearing | Non-weight-bearing standing and walking |
| 3 | Poor | Partial weight-bearing standing; non-weight-bearing walking |
| 2 | Acceptable | Partial weight-bearing standing and walking |
| 1 | Good | Normal standing: favours affected limb when walking |
| 0 | Excellent | Equal on all limbs standing and walking |
